# Cortical Metabolic Arrangement During Olfactory Processing: Proposal for a ^18^F FDG PET/CT Methodological Approach

**DOI:** 10.1097/MD.0000000000000103

**Published:** 2014-10-17

**Authors:** Alessandro Micarelli, Marco Pagani, Agostino Chiaravalloti, Ernesto Bruno, Isabella Pavone, Matteo Candidi, Roberta Danieli, Orazio Schillaci, Marco Alessandrini

**Affiliations:** Department of Clinical Sciences and Translational Medicine (AM, EB, IP, MA), Tor Vergata University; Institute of Cognitive Sciences and Technologies-CNR (MP), Rome, Italy; Department of Nuclear Medicine (MP), Karolinska University Hospital, Stockholm, Sweden; Department of Biomedicine and Prevention (AC, RD, OS), Tor Vergata University; Department of Psychology (MC), “Sapienza” University, Rome; and IRCCS Neuromed (OS), Pozzilli, Italy.

## Abstract

The aim of this article is to investigate the cortical metabolic arrangements in olfactory processing by using ^18^F fluorodeoxyglucose (FDG) positron emission tomography/computed tomography.

Twenty-six normosmic individuals (14 women and 12 men; mean age 46.7 ± 10 years) were exposed to a neutral olfactory condition (NC) and, after 1 month, to a pure olfactory condition (OC) in a relatively ecological environment, that is, outside the scanner. All the subjects were injected with 185–210 megabecquerel of ^18^F FDG during both stimulations. Statistical parametric mapping version 2 was used in order to assess differences between NC and OC.

As a result, we found a significant higher glucose consumption during OC in the cuneus, lingual, and parahippocampal gyri, mainly in the left hemisphere. During NC, our results show a relative higher glucose metabolism in the left superior, inferior, middle, medial frontal, and orbital gyri as well as in the anterior cingulate cortex.

The present investigation, performed with a widely available functional imaging clinical tool, may help to better understand the neural responses associated to olfactory processing in healthy individuals and in patients with olfactory disorders by acquiring data in an ecologic, noise-free, and resting condition in which possible cerebral activations related to unwanted attentional processes might be avoided.

## INTRODUCTION

The sense of smell in human and animals is one of the chief sensory systems that allow connecting with the environment through a complex chemosensory process.

Data from neuroimaging studies suggest that the sense of smell is strictly dependent on the specific anatomo-functional characteristics of the systems where early olfactory signal processing takes place, namely, limbic and paralimbic structures.^1^

Anatomically, the olfactory system is unique: it is characterized by direct connections between the external environment (olfactory receptor cells) and the brain (first synapse, the olfactory bulb). Sensory information is delivered to the cerebral cortex without an initial relay to the thalamus^2^ and cortical olfactory areas are phylogenetically older than other sensory cortical areas showing a different organization of their layers (allocortex vs isocortex).

Before reaching their central projection loci, odorant stimuli are processed through 3 inherently different systems involving the olfactory tract, the trigeminal nerve, and the vomeronasal organ (VNO). The relative involvement of each of these systems depends on the nature of the olfactory compound and, whereas most odorants act through both the olfactory and the trigeminal nerves, pure odorants such as vanilla (VAN) or lavender activate only the olfactory nerve.^1^ The combined activation of the olfactory tract and the trigeminal nerve, involving responses to compounds such as acetone and butanol, can lead to a burning sensation. The third system, involving the VNO, is thought to respond to pheromones.^1^

The human olfactory system, similar to other sensory ones, must decipher both the identity and the intensity of perceived stimuli, and along the path, olfactory processing occurs at several levels from the periphery to the brain. Thus, higher order processing regions integrate information from sensory neurons with associational and state-dependent cues in order to drive behavior.^3,4^ In addition to these variables, literature on olfaction highlighted 2 other important issues. The first one concerns the perception and the sensation of smell: in fact, these 2 phenomena are largely dependent on sniffing behaviors that may be modulated by attentional mechanisms in order to increase the probability of detecting odors. Indeed, the subsystems controlling sniffing and smelling are separated in the human olfactory cortex and the different airflows they produce could result in asymmetries in cerebral activations.^5^ The second important issue concerns the chemosensory inputs that can automatically induce an unwanted hedonic (emotional) response, implying an involvement of the limbic system. In fact, when a pure odorant compound, such as VAN, is used, H_2_^15^O-positron emission tomography (H_2_^15^O-PET) scanning shows projections not only to the olfactory bulb but also to the limbic system, for example, to the piriform, orbitofrontal, and anterior cingular cortices and the agranular insular region.^1^

The existing literature on changes in brain activation during olfactory tasks is mainly based on neuroimaging techniques that unify the stimulation and the acquisition phases in the camera gantry often perceived as an unfriendly, noisy, and not odorless environment (ie, functional magnetic resonance imaging [fMRI] shows strong unintended auditory and olfactory stimulation through the period of experimental stimulation and data acquisition), constituting a common bias to the interpretation of the majority of existing data.^6,7^ Furthermore, MRI examinations are often difficult if not impossible to stand for many patients with neurodegenerative, psychiatric, and emotional disorders. Conversely, ^18^F fluorodeoxyglucose (FDG)-PET/computed tomography (CT) is a functional neuroimaging methodology that allows the investigation of the biochemical changes coupled to the cerebral glucose metabolism in relatively ecological environments,^6,8^ that is, in a comfortable, quiet, light, and airy room, avoiding possible biases resulting from physical and psychological discomfort for the patient.^6,7^ For these reasons, it seems important to report the metabolic changes associated to an olfactory stimulation under these conditions. Furthermore, FDG-PET/CT wide availability in clinical environments and the relatively standardized image acquisition protocols favors the reproducibility of studies.

To the best of our knowledge, to date only 1 study investigated the olfactory neural correlates in a resting-state condition by using FDG-PET/CT.^9^ In fact, the majority of imaging studies concerning the neural responses to an olfactory stimulation used techniques such as fMRI and H_2_^15^O-PET/CT, and are thus dependent on the presence of an in-house cyclotron along with a complicated and sensitive methodology, which may not be optimal for clinical studies.

In the present study, we aimed at assessing the cortical metabolic involvement to a pure olfactory stimulation by using FDG-PET/CT in a larger cohort of normal subjects at rest.^9^ The findings may contribute to describe the cerebral responses to olfactory stimulations.

## MATERIALS AND METHODS

### Subjects

Twenty-six right-handed individuals (14 women and 12 men; mean age 46.7 ± 10 years) without otorhinolaryngologic or neurological diseases were enrolled in the study. All of them were assessed as normosmic when evaluated with the multiple-forced-choice Sniffin’ Sticks screening test.^10^ A detailed case history was collected for all subjects who underwent ear–nose–throat examination with fiberoptic examination of the upper airways. Neurological diseases were excluded with the mini-mental state examination and MRI. Considered as exclusion criteria were all those conditions that could potentially develop an olfactory dysfunction, that is, sinonasal disorders or surgery history, head trauma, neuropsychiatric disorders (Parkinson disease, Alzheimer disease, schizophrenia, multiple sclerosis, depression, and multiple chemical sensitivity/idiopatic environmental intolerance), lower airways and/or lung diseases, active hepatitis, cirrhosis, chronic renal failure, vitamin B12 deficiency, alcohol, tobacco or drug abuse, cerebral vascular accidents, insulin-dependent diabetes mellitus, hypothyroidism, and Cushing syndrome.

Moreover, we excluded all patients in treatment with drugs that could interfere with ^18^F FDG uptake and distribution in the brain.^11^ No patients were pregnant or breastfeeding and all participants signed a written informed consent form according to the principle outlined in the Declaration of Helsinki.

### Experimental Procedure

All 11 subjects underwent FDG-PET/CT after a neutral olfactory stimulation by using a common aerosol facial mask containing in its ampoule only 5 mL of saline sodium chloride (NaCl) 0.9% (neutral olfactory condition [NC]; n = 26). After 1 month, they underwent a second FDG-PET/CT scan after a simple olfactory stimulation by using the same aerosol facial mask containing in its ampoule a solution of 1.5 mL of VAN 100% and 5 mL of saline NaCl 0.9% (pure olfactory condition [OC]; n = 26). In both the conditions, oxygen flow rate was conveyed at 3.5 L/min and the stimulation consisted of one continuous 9 minutes block without any sniffing-generated instructions. At the end of the third minute, each subject was injected with 185–210 megabecquerel (mBq) of ^18^F FDG intravenous and the olfactory stimulation continued for 6 more minutes. After both NC and OC, all subjects laid down in a semidarkened, noiseless, and odorless room, without any artificial stimulation, with their eyes closed for 20 minutes.

### PET/CT Scanning

The PET/CT system Discovery ST16 (GE Medical Systems, Powell, TN) was used. This system combines a high-speed ultra 16-detector-row (912 detectors per row) CT unit and a PET scanner with 10,080 bismuth germanate crystals in 24 rings with a 128 × 128 matrix. Crystal size 6.2 × 6.2 × 30 mm, coincidence window 11.7 nanoseconds, system sensitivity 9.3 cps/kBq in 3D mode, dispersion fraction 44%, maximum count rate in cps at 50% dead time 63 kcps @ 12 kBq/mL (3D), axial full width at half maximum (FWHM) 1 cm radius 5.2 mm in 3D mode, and axial field of view 157 mm.

Before and after FDG injection, hydratation (500 mL of iv NaCl) 0.9%) to reduce the pooling of the radiotracer in the kidneys was performed. All subjects had normal serum glucose level and fasted for at least 5 hours before the ^18^F FDG injection.^12^

### Statistical Analysis

Differences in brain FDG uptake were analyzed using statistical parametric mapping (SPM2, Wellcome Department of Cognitive Neurology, London, UK) implemented in Matlab 6.5 (Mathworks, Natick, MA). PET data were subjected to affine and nonlinear spatial normalization into the Montereal Neurological Institute space. The spatially normalized set of images were then smoothed with a 8-mm isotropic Gaussian filter to blur individual variations in gyral anatomy and to increase the signal-to-noise ratio. Images were globally normalized using proportional scaling to remove confounding effects to global metabolic changes, with a threshold masking of 0.8. The resulting statistical parametric maps (SPMt) were transformed into normal distribution (SPMz) unit. Correction of SPM coordinates to match the Talairach coordinates was achieved by the subroutine implemented by Matthew Brett (http://www.mrc-cbu.cam.ac.uk/imaging). Brodmann areas (BAs) were then identified at a range of 0 to 3 mm from the corrected Talairach coordinates of the SPM output isocentres, after importing them by Talairach client (http://www.talairach.org/index.html). A statistical height thresholds equal or lower than *P* < 0.05 at both cluster and voxel level was accepted as significant. This more liberal choice was adopted to avoid type II errors attributable to overconservative thresholds.^13^ Effectively, given the exploratory nature of this analysis and considering the relatively low sensitivity of PET without repeated measures, higher thresholds could lead to false-negative results in PET studies. Only those clusters containing more than 125 (5 × 5 × 5 voxels, ie, 11 × 11 × 11 mm) contiguous voxels were accepted as significant, based on the calculation of the partial volume effect resulting from the spatial resolution of the PET camera (about the double of FWHM).

The OC versus NC comparisons were performed by means of the “compare populations: 1 scan/subject (Ancova)” option, using age and sex as covariates.

## RESULTS

**FIGURE 1:**
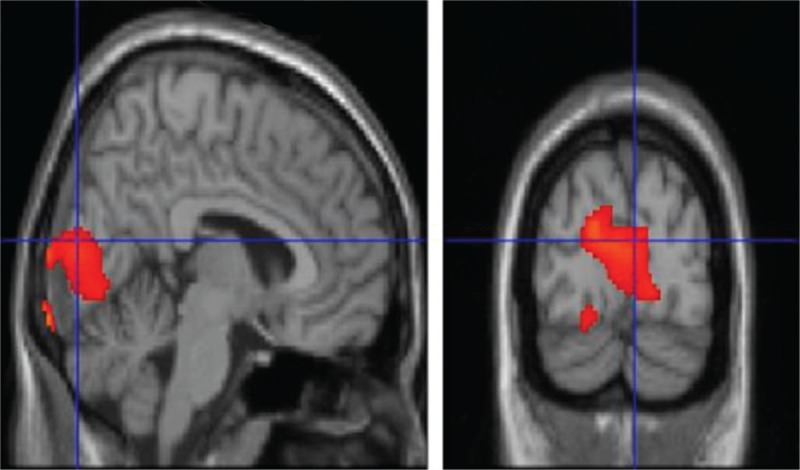
T1 MRI superimposition showing the cluster of voxels in the cuneus, lingual gyrus, and parahippocampal gyrus, mainly in the left hemisphere, in which FDG uptake was significantly higher at OC (n = 26) as compared to NC (n = 26) (on the left sagittal and on the right coronal projections). Coordinates and regional details are presented in Table 1. FDG = ^18^F fluorodeoxyglucose, MRI = magnetic resonance imaging, NC = neutral olfactory condition, OC = pure olfactory condition.

**FIGURE 2:**
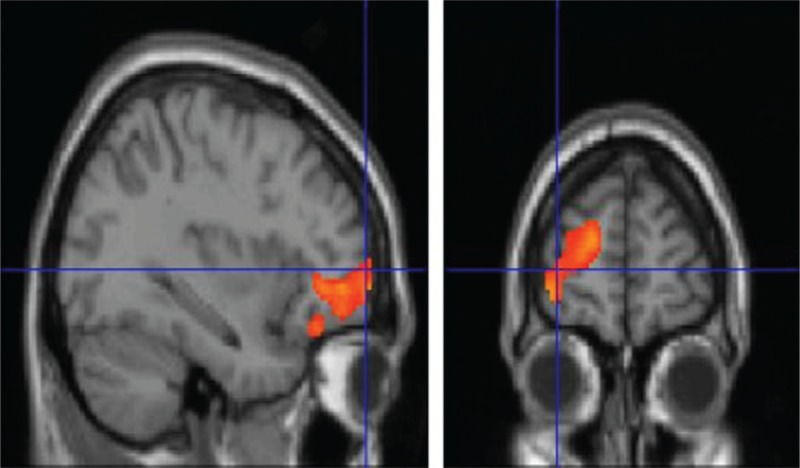
T1 MRI superimposition showing the cluster of voxels in the left orbitofrontal and anterior cingulate cortex in which FDG uptake was significantly higher at NC (n = 26) as compared to OC (n = 26) (on the left sagittal and on the right coronal projections). Coordinates and regional details are presented in Table 2. FDG = ^18^F fluorodeoxyglucose, MRI = magnetic resonance imaging, NC = neutral olfactory condition, OC = pure olfactory condition.

A significantly higher glucose metabolism was found in OC compared to NC in cuneus (CU), lingual gyrus (LG), and parahippocampal gyrus (PHG), mainly in the left hemisphere (Table 1, Figure 1). In the opposite comparison (NC compared to OC), a relative higher glucose metabolism was found in left superior, inferior, middle, medial frontal, and orbital gyrus (orbitofrontal cortex [OFC]) as well as in anterior cingulate cortex (ACC) (Table 2, Figure 2).

**TABLE 1:**
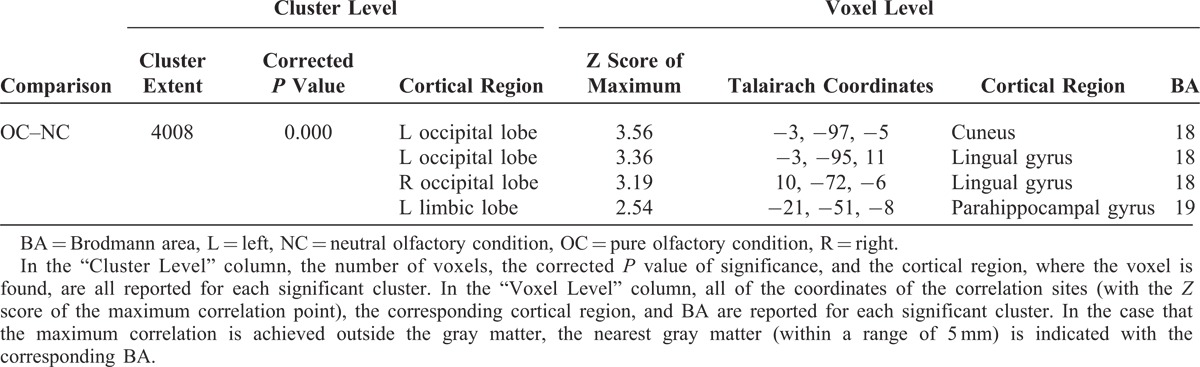
Numerical Results of SPM Comparisons Between ^18^F FDG Uptake in OC and NC (n = 26)

**TABLE 2:**
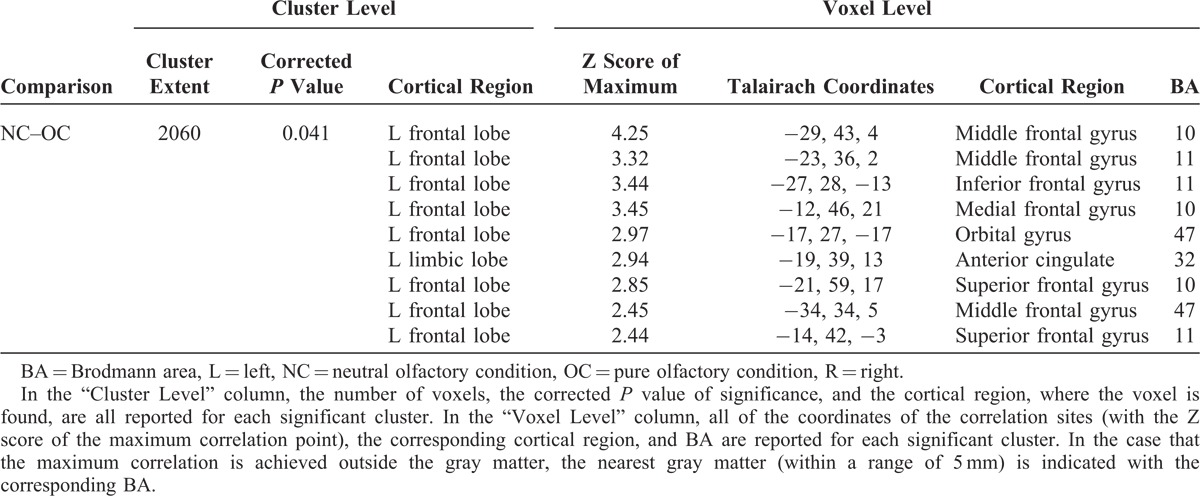
Numerical Results of SPM Comparisons Between ^18^F FDG Uptake in NC and OC (n = 26)

## DISCUSSION

A review of the literature since the original work of Zatorre et al^14^ in 1992 to now shows that many functional and structural imaging techniques were applied to study the olfactory system using a number of different olfactory tasks. Neural correlates of olfaction, both in healthy and pathological subjects, have been studied using a variety of methods such as fMRI,^15,16^ voxel-based morphometry (VBM)^17^ and H_2_^15^O-PET.^18–21^ However, because of the heterogeneity of methods used across the studies, the interpretation and comparison of results is often difficult. Indeed, different studies showed to be different along the emotional continuum, number of stimuli presented, tasks performed by the subjects, population in study, and imaging techniques. More importantly, here, a common problem to these earlier studies concerns the environmental conditions in the scanner. More importantly, here, a common problem to these earlier studies concerns the environmental conditions in the scanner with the subject being often positioned in a noisy, not odorless, camera.

Thus, it remains unclear to what extent discrepancies in the results of different studies may reflect different sensory-specific engagements in inducing olfactory perception. On the contrary, variability in magnitude, laterality, or specific location of responses may reflect sensory-specific modulations rather than methodological differences. For instance, it is unknown whether different pleasant and unpleasant odorants (presented and controlled in different ways) engage common cortical and subcortical regions or different discrete sensory-specific subregions.^18^

Nevertheless, many neuroimaging studies showed that the primary olfactory cortex—encompassing distinct regions as the piriform and entorhinal cortex, amygdala, anterior olfactory nucleus, and olfactory tubercle^22^—is intimately connected to secondary olfactory centers.^14–20^

When considering these aspects, many results support the idea that the neural correlates of odor processing are task dependent, involving a distributed network of structures—even outside core olfactory regions—determined by the nature of the context and the specific olfactory task at hand.^23^

In agreement with the previous cited report of Alessandrini et al,^9^ we found an higher glucose uptake in CU, LG, and PHG mainly in the left hemisphere, when comparing OC to NC. These activation were previously undiscovered and have been highlighted here by using a FDG-PET approach (Table 1, Figure 1).

An olfactory-related parahippocampal involvement was indirectly demonstrated by Bitter et al^17^ in a VBM approach study in which the authors described that subjects with anosmia of different etiology had gray matter reductions in primary as well as secondary olfactory regions such as PHG. More recently, Kjelvik et al^24^ reported brain activations related to a passive smelling model by using a fMRI approach. They highlighted that brain activity in relation to spontaneous odor identification (OI) is distinct from that associated to nonidentified odors, and also differs from activity during passive smelling. OI specifically increased the activity in the entorhinal cortex and hippocampus, as well as in the PHG. Furthermore, several studies describing olfactory neural networks, using a wide variety of odors and tasks, have reported hippocampal and PHG activity, although lateralization and location along the anterior–posterior axis of the hippocampus varies across the literature.^16,25–27^

Our study showed a relative OC-related higher glucose uptake in the PHG; this finding is in accordance with the familiarity-based recognition idea assessed in the literature, along with the anatomical and connectivity characteristics of the medial temporal lobe, suggesting that the parahippocampal cortex may encode representations of the global context in which an item was encountered^28–30^ and the increase in activity in this region may reflect an increased reliance on perceptual fluency during familiarity-based recognition.^31^

The second important aspect of the present study is the relative hypermetabolism in OC condition found in CU and LG areas of the visual cortex. This finding is consistent with previous studies reporting the visual cortex to be involved in visual–olfactory interaction and activated, for instance, by identifying objects and generating mental visual images.^32^ The activation of the occipital cortex is a common finding in olfactory neuroimaging and electrophysiological studies^16,25,32–38^ for which one speculative explanation is that once subjects identify the olfactory stimulus, based entirely on processes occurring within olfactory brain regions, they tend to visualize and conceptualize the corresponding object. In this case, visual cortical activity might be correlated with olfactory perception via imagery processes, but it would have no influence over the percept itself.^37,39^

Interestingly, the relative hypometabolism in OFC and ACC (Table 2, Figure 2) when comparing OC with NC is an intriguing aspect that expands current knowledge on the specific activity of such regions, implicated in many olfactory tasks. Indeed, OFC—an area reported to process common odors^27,33,40^—showed a relative decreased glucose uptake when comparing OC with NC. Gottfried and Zald^41^ showed that lateral and anterior regions of the OFC responded in a preferential manner to binary odor mixtures. By investigating regional cerebral blood flow in the lateral and anterior OFC, they found these 2 regions to respond to mixtures in different ways. Specifically, activation in the lateral OFC increased with increasing odorant impurity, as indicated by an inversed U-shaped function peaking at the most impure mixture. On the contrary, the anterior region of the OFC was equally activated by all binary odor mixtures and deactivated by the single odors.^41^ Interestingly, these data were further confirmed in the study performed by Boyle et al^21^ in which they found that the anterior OFC acted as a sort of on–off switch for which this region was similarly activated in response to all odor mixtures and deactivated in response to single odorants.

To date, all the studies that reported activation in the anterior OFC in response to single pure olfactory stimuli were performed by using fMRI and H_2_^15^O-PET/CT.^5,24,42^ Although, as compared to fMRI and H_2_^15^O-PET/CT, the temporal resolution of brain activation as recorded by FDG is inferior, in our study, we could replicate for the first time the results previously found via these 2 techniques. This was also because of the robust within-subjects experimental model allowing fairly good statistics in relation to the number of investigated subjects. Our findings suggest that FDG might serve as a suitable imaging modality for investigating brain cortical activation/deactivation during olfactory tasks.

For clinical studies, in fact, the brain tissue can be considered as a 3-compartment model^43–45^ where the tissue is homogeneous with respect to rate of blood flow rates of transport of glucose and deoxyglucose (DG) between plasma and tissue, concentrations of DG, glucose, and deoxyglucose-6-phosphate (DG-6-P), and rate of glucose utilization. DG-6-P, once formed, is essentially trapped in the tissue for a reasonable time (as the time after the injection in our experimental procedure) allowing to obtain images of the FDG kinetics in brain after the injection.^43–45^ In particular, considering the trend “en plateau” of the kinetic of FDG, 40% of the radiolabeled compound is extracted in the brain (∼250 nCi/g) in the first minutes after the injection, thus allowing the detection of the cortical brain areas that are activated in the first timings of our task.^43–45^ Interestingly, in resting conditions, the rate of brain glucose utilization does not show significant changes over time. In the cited study of Sokoloff et al,^45^ in all the examined rats’ brain areas, the 14 C DG concentrations did not show significant changes over time being 104 ± 2 and 98 ± 5 μmol/mL, respectively, in the early and delayed assays in the primary olfactory cortex.

On the contrary, the improved sensitivity of the state-of-the-art PET cameras allows to detect reliable signal changes also under suboptimal condition. The possibility to create a defined ecologic baseline condition to the exploration of olfactory neural underpinnings helped in avoiding possible cortical activation related to unwanted attentional processes due to the examination environment. This increases the dynamic range of brain metabolism as an informative correlate of neural activity. Thus, this notion of baseline implies that during a particular task, not only activation is observed but also deactivation in certain areas is found,^8^ and depicts an organized default mode network (DMN)^46^ in which some regions are most active during resting state.^46–48^ These regions have been thought to be involved in monitoring the internal and external milieu^46–50^ and an emerging point is that the DMN is composed of a group of relatively large and interconnected areas, including the ACC and ventromedial prefrontal cortex (BA 10).^46^ Hence, when introducing a pure odor compound in the present experimental model, a cortical reorganization pattern from anterior (OFC and ACC in NC) to posterior (CU, LG, and OHG in OC) regions was in line with Magistretti’s observations. This specific neural behavior could suggest, for the first time, a possible rearrangement of cortical activity when experimenting a pure and passive olfactory stimulation condition. To this end, the CU hub network, including LG, has been previously found to be negatively correlated with DMN activity, suggesting that an anticorrelated activity between the former regions and the second network could be engaged by sensory processing tasks.^51^ Finally, although previous olfactory-related imaging studies have demonstrated a bilateral activation in primary olfactory cortex and greater activation in the right than in the left orbitofrontal cortex,^23,25,52^ in the present study, we found a global leftward asymmetry both when comparing OC to NC and vice versa. Possible explanation for this inconsistency could be related to the experimental model setting in which any retrieval/recognition task of the incoming was generated. Regarding these aspects, a switching of lateralization from right to the left hemisphere associated to a switch from recognition to semantic processing of the online information, for both verbal and nonverbal materials, has been widely assessed by the hemispheric encoding/retrieval asymmetry model.^53^

## CONCLUSIONS

As a rule, olfaction always forms one component of multisensory events; therefore, further investigations are widely appreciated for a thorough understanding of the cortical involvement during olfactory processing. We believe that the present findings, together with previous studies, might help in better understanding the association between neurological and psychiatric diseases in which olfactory disorders are observed and disruption of connectivity in the DMN and cortical lateralization coexist.

Moreover, the proposed olfactory-related FDG-PET/CT approach, in a relatively ecological environment, suggests that the technique might enjoy a wide spread, because of its ease and PET/CT scans diffusion.
